# Treatment Options for Distal Rectal Cancer in the Era of Organ Preservation

**DOI:** 10.1007/s11864-024-01194-4

**Published:** 2024-03-22

**Authors:** Chen Wang, Xiaoliang Liu, Weiping Wang, Zheng Miao, Xiaoyan Li, Dingchao Liu, Ke Hu

**Affiliations:** Department of Radiation Oncology, Peking Union Medical College Hospital, Chinese Academy of Medical Sciences & Peking Union Medical College, NO.1 Shuaifuyuan Wangfujing, Dongcheng District, Beijing, 100730 China

**Keywords:** Distal rectal cancer, Organ preservation, Adjuvant radiotherapy, Adjuvant chemotherapy, Watch and wait, Radical radiotherapy

## Abstract

The introduction of total mesorectal excision into the radical surgery of rectal cancer has significantly improved the oncological outcome with longer survival and lower local recurrence. Traditional treatment modalities of distal rectal cancer, relying on radical surgery, while effective, take their own set of risks, including surgical complications, potential damage to the anus, and surrounding structure owing to the pursuit of thorough resection. The progress of operating methods as well as the integration of systemic therapies and radiotherapy into the peri-operative period, particularly the exciting clinical complete response of patients after neoadjuvant treatment, have paved the way for organ preservation strategy. The non-inferiority oncological outcome of “watch and wait” compared with radical surgery underscores the potential of organ preservation not only to control local recurrence but also to reduce the need for treatments followed by structure destruction, hopefully improving the long-term quality of life. Radical radiotherapy provides another treatment option for patients unwilling or unable to undergo surgery. Organ preservation points out the direction of treatment for distal rectal cancer, while additional researches are needed to answer remaining questions about its optimal use.

## Introduction

Distal rectal cancer is usually defined as cancer which is located within 5–6 cm from the anal margin or at the lower third of the rectum more than 2 cm above the dentate line. Compared with high rectal cancer, the peculiar lateral lymphatic drainage pathway in low rectal cancer, which may cause lymph node metastasis, increases the risk of recurrence. Total mesorectal excision (TME) has markedly improved the oncological outcome during the past decades. However, operation-related organ loss and functional impairment because of the anatomical limitations of a narrow pelvis and operative difficulty impair the quality of life (QoL) for patients and affect social function and psychological health status [1]. Therefore, patients and doctors have paid more and more attention to organ preservation strategy. Organ preservation for distal rectal cancer is designed to ensure both the best oncologic and functional results without a permanent stoma. The ideal treatment promises not only adequate circumferential resection margin but also functional sphincter muscles. Organ and function preservation strategy has been wildly used, benefitting from the development of comprehensive examining methods, improvement in operational styles, and application of adjuvant or neoadjuvant chemoradiotherapy. This article aims to summarize the progress in organ and function preservation in distal rectal cancer.

## Operating methods

Abdominoperineorectal resection (APR), the standard treatment for distal rectal cancer in the early twentieth century, ensured distal and lateral border safety, but had a poor prognosis and high recurrence rate [2]. Then TME was proposed in the late twentieth century, significantly reducing the local recurrence rate (LRR), and has become the gold standard for radical rectal cancer surgery. However, the permanent fistula and the loss of anal function after radical surgery have a great impact on QoL and mental health. In order to improve the long-term QoL and psychological state of patients of rectal cancer, a variety of anal preservation methods were proposed. The ideal organ preservation therapy for distal rectal cancer maintains the structure and function of the sphincter muscle of the anus by local excision (LE) without sacrificing oncologic outcome.

### Low anterior resection, LAR

LAR is currently the most classic and used surgical method for distal rectal cancer, and it is also the preferred surgical method for distal rectal cancer. On the basis of meeting the principle of TME, LAR preserves the complete structure of the anal sphincter, anal canal, and various receptors in the anal canal, thus preserving the complete anal defecation function after surgery. An analysis based on data from the US Cancer Data Center from 2010 to 2015 showed that laparoscopic, robotic, and open surgery had slight differences in resection margin status, length of stay, readmission, and overall survival, but the results were all acceptable considering selection bias [3]. In addition to the selection of surgical methods, the relevant researches focus on the selection of anastomosis and postoperative reconstruction methods [4].

### Intersphincteric resection, ISR

ISR selectively excised the internal rectal sphincter while preserving the external rectal sphincter, while preserving the functional tissue associated with the rectum to preserve postoperative anal function. It expands the chance of anal preservation in very low rectal cancer. It applies to patients with stage T1-2 or T2 infiltrating the internal sphincter and patients with stage T3 and neoadjuvant therapy completed. Or the lesion has a sufficient distance from the anus but with a narrow pelvis difficult to perform pelvic anastomosis. According to the excision range of the internal sphincter, ISR can be divided into partial ISR, subtotal ISR, and complete ISR, in which 1/3 ~ 1/2, 2/3, and the whole internal sphincter are excised, respectively [5]. Considering that the internal sphincter provides 50–85% of the resting pressure of the anal canal, partial removal of the internal sphincter in ISR will inevitably lead to a decrease in the resting pressure of the anal canal, resulting in decreased fecal control. An observational study in Japan compared the long-term anal function after ISR and LAR. Wexner score in the LAR group increased 6 months after surgery, but recovered to the pre-treatment level 12 months after surgery. Although the Wexner score in the ISR group was higher than that before surgery, it decreased significantly 24 months after surgery compared with 6 months after surgery. This suggested that anal function gradually improves over time [6]. A 5-year follow-up of patients after ISR in Japan showed good OS and few treatment-related complications after ISR. However, the LRR was high, remaining 11.5% in 5 years. Stratified by the T stage, the LRR of pT3 was 18.1%, and 36.0% for pT4, which was significantly increased compared with 8.8% in previous studies. In addition, postoperative fecal incontinence, severe bowel dysfunction, and severe urinary incontinence are very common [7]. Therefore, for patients undergoing ISR, preoperative screening should be carefully performed to avoid serious postoperative dysfunction and inability to achieve actual anal preservation.

### Transanal endoscopic microsurgery, TEM

In 1983, Gerhard Buess from Germany invented the TEM technique. One hundred forty patients with adenomas were treated, 30 proved to be carcinoma part of whom receiving radical reoperation [8]. Long-term follow-up results justify TEM alone as curative treatment in low-risk rectal carcinoma, including pT1, G1/2, L0, and LX with clear margins and a minimal distance between tumor and resection margin over 1 mm [9]. With low recurrence rates and cancer-related death, TEM has a similar therapeutic effect to radical surgery in early low-risk rectal cancer. As for high-risk rectal cancer, the high local recurrence rate restricts the use of TEM alone as a compromise therapy [10].

### Transanal minimally invasive surgery, TAMIS

The indications of TAMIS for early rectal cancer are similar to TEM, including T1 stage rectal cancer that invades submucosa less than 1/3 and has good pathological features [11]. TAMIS has comparable short-term and long-term efficacy as TEM in early rectal cancer, with cumulative DFS recorded in 96%, 93%, 84%, and 78% at 1, 2, 3, and 5 years. Postoperative morbidity was noted in 9–11% of patients [12–14]. And because the equipment installation time is omitted, TAMIS is associated with shorter operative time and length of stay [12, 15]. Owing to the relatively short application time of TAMIS, the comparison of its efficacy with TEM needs to be confirmed by large-scale and prospective clinical studies.

### Transanal total mesorectal excision, TaTME

TaTME is a new surgical technique proposed based on the concept of natural orifice transluminal surgery (NOTES) for middle and low rectal cancer. This technique is suitable for patients with preoperative stage ≤ T3 and small tumor volume. It has a prominent advantage in male or obese patients or those with narrow pelvis. Multiple systematic reviews and meta-analyses suggested that TaTME has no significant difference or is even better compared with laparoscopic total mesorectal excision (LaTME) in terms of overall and major morbidities, anastomotic leak, readmission rate, CRM involvement, and length of stay [16, 17]. TaTME is relatively complex and requires high technical requirements for surgeons, so whether it can be widely used is still controversial. Suspension of TaTME in Norway is an example. Studies show that TaTME can be safely implemented under supervision and quality assurance [18]. A randomized, open-label, phase 3, non-inferiority trial performed at 16 different hospitals in China included 1115 patients of low rectal cancer staging within cT3N2. They were randomized 1:1 to receive TaTME or LaTME by experienced surgeons [19]. There were no significant differences between the two groups in short-term outcomes including intraoperative complications, postoperative morbidity, mortality, and successful resection. The long-term outcomes of local recurrence and distant metastasis need to be further followed up.

## Local excision followed by adjuvant therapy in early rectal cancer

### Adjuvant radiotherapy—external beam radiotherapy

As a supplementary to early rectal cancer with a high risk after LE, adjuvant radiotherapy preserves the advantages in organ preservation. Several studies suggest that adjuvant radiotherapy can be used as an alternative to radical surgery for early rectal cancer with high-risk factors after LE. A retrospective single-center study in Canada investigated the efficacy of adjuvant radiotherapy (Dt 40–50.4Gy) after LE in 93 patients with T1-3N0 rectal cancer between 2001 and 2010 [20]. The 5-year OS, local control (LC), and progress-free survival (PFS) were 78.5%, 86.1%, and 83.8%, respectively, showing good level of local control in T1 disease and good treatment option for patients who are either medically not suitable for a more radical surgical approach or who refuse this procedure. A recent retrospective study based on the SEER database analyzed 3786 patients with T2N0M0 rectal cancer from 1998 to 2013. A total of 81.0% of these patients received radical surgery, 11.3% received simple LE, and 7.7% received LE followed by adjuvant radiotherapy. The 5-year CSS rates were 81.8%, 70.5%, and 78.4%, and the 5-year OS rates were 72.3%, 57.3%, and 70.7%, respectively [21]. A prospective analysis from Spain included 88 patients with T1–2 stage rectal cancer who underwent LE and 28 patients with high-risk pT1 or low-risk pT2 [22]. Patients received adjuvant radiotherapy of 50.4 Gy/28f after LE, the 6-year cancer-specific survival (CSS) was 93%, and three patients (10.7%) had disease recurrence. Another prospective study included 14 patients with high-risk factors who received adjuvant radiotherapy after local resection, with a 5-year OS of 78.6% and a 5-year DFS of 85.7%, suggesting that adjuvant radiotherapy can also achieve a satisfactory survival outcome for those who cannot tolerate the combination of adjuvant radiotherapy and chemotherapy [23]. However, the systematic review by Borstlap presented a high recurrence rate after transanal excision and adjuvant (chemo)radiotherapy [24]. The risk of local disease recurrence after adjuvant therapy (14%) was higher than that after radical surgery (7%). In contrast, these two treatments had the same distal metastasis (DM) of 9%, 5-year OS of 61–80% and 79–100%, and 5-year DFS of 75–100% and 94%, respectively. The undesirable recurrence rate may be affected by the following factors: (1) the consecutive enrollment of the group with radical therapy but not the group with adjuvant therapy, (2) differences in patients’ baseline conditions, (3) no N-stage stratified treatment, and (4) follow-up arrangement: some studies did not state the plan, and local disease recurrence in the group with adjuvant treatment may miss the best time for salvage treatment.

### Adjuvant radiotherapy—brachytherapy

To reduce the risk of disease recurrence at the site of endoluminal resection, brachytherapy is another option for patients at high risk of disease recurrence after LE. A retrospective study from the UK analyzed 180 rectal cancer cases staging pT1-T3 that received brachytherapy after local resection of rectal cancer, most of which were combined with external beam radiation therapy (EBRT) or concurrent chemoradiotherapy (CRT) [25]. The 36-month follow-up showed a local disease recurrence-free rate of 94%. The CONTEM1 study conducted a longer follow-up in patients with high risk after LE, who received Papillon50tm 40–60 Gy/2–3 f/2–4 w of brachytherapy, with or without EBRT [26••]. The 6-year LRR is 8%, with DM of 9%, local disease recurrence-free survival rate of 91%, OS of 81%, CSS rate of 97%, no treatment-related deaths, and organ preservation rate of 95%. The LRR was 4% with brachytherapy before EBRT and 15% afterward, suggesting that the timing of brachytherapy is related to efficacy. For patients who cannot tolerate radical surgery and concurrent CRT, brachytherapy can achieve favorable outcomes as a relatively mild treatment. Currently, there are no direct comparisons with other treatment options. Further randomized trials will verify whether it can achieve the same results as concurrent CRT.

### Adjuvant chemoradiotherapy

Adjuvant radiotherapy combined with chemotherapy has a potential radiosensitization effect, reducing the incidence of adverse reactions and improving the LC. Sasaki et al. [27] conducted a prospective, single-arm, phase II multicenter study, including 57 patients after LE identified as T1N0 with a risk factor for lymph node melanomametastasis or T2N0 rectal cancer located below the peritoneal reflection. These patients received additional EBRT of 45 Gy plus continuous 5-week intravenous injection of 5-fluorouracil (250 mg/m^2^ per day). With 7.3 years median follow-up, the 5-year DFS rate was 94% for T1 lesions, 75% for the T2 lesions, and 2 local recurrences during the observation period. The short-term adverse reactions were mainly skin damage, anal pain, and anal mucositis, grade 2 or below, with a few grade 3 reactions, and late treatment-related adverse reactions were rare. The retrospective study of Li et al. [28•] included intermediate-risk patients with early rectal cancer after TEM from 2010 and 2017. The intermediate risk was defined as pT1 with a large diameter (3–5 cm), lymphovascular invasion, or poor differentiation or pT2 with a small diameter (< 3 cm). Adjuvant CRT (50.4 Gy + FOLFOX/Xelox/Capox) after LE presented comparable OS and DFS to that for TME surgery, higher but not significant for LRR as 9.1% vs 5.4%, respectively. A prospective study by Javed et al. [23] compared different treatment strategies for patients with poor prognostic indicators after local resection. Of the 53 patients, 18 had TME, 14 had CRT, 14 had RT, and 7 had no further treatment. Compared to 8 recurrences and 2 deaths in the surgical group, there were no recurrences or deaths in the CRT group. These findings support the strategy of adjuvant CRT as an alternative treatment to radical surgery for patients of ERC with poor prognostic factors after LE. The specific efficacy and long-term safety of adjuvant therapy after LE remain unknown due to limited follow-up time. The ongoing TESAR study [29] is the first multicenter randomized trial in which patients with intermediate-risk T1-T2 rectal carcinoma after LE will be randomized to rectal-preserving adjuvant CRT or radical surgery. This trial will further illustrate the role of organ preservation in tumor outcomes, functional preservation, and quality of life, providing guidance in the management of patients with high risk after LE.

## Neoadjuvant therapy

### Neoadjuvant therapy

Total neoadjuvant therapy (TNT) combined with total mesorectal excision (TME) is currently the standard treatment for stage T3-T4/N + locally advanced rectal cancer (LARC) [30, 31]. This regime eliminates tumor micrometastases at the early stage and improves patient compliance with treatment [32•]. After radiotherapy-centric neoadjuvant treatment, about 60% of rectal cancer regresses or downstages and 15–30% even achieves pathological complete response (pCR), with 5-year survival rates over 90% and rare local recurrence [33–36]. For this group of patients, the significance of radical resection for local control has been greatly weakened. Since 2004, several studies focused on patients achieved clinical complete response (cCR) after neoadjuvant and W&W therapy has been performed. Compared with patients with pCR after radical surgery, patients with cCR shared similar survival rate and local control rate however preserved anal structure, thus remarkably improved the anal function and quality of life [37–41]. This discovery illuminates the possibility of organ and function preservation strategy for more patients.

The optimization of neoadjuvant therapy is a heated topic at present, aiming to improve cancer response which may improve survival. The highlights of the discussion include fractionation of radiotherapy, sequence of chemoradiotherapy, intensity of neoadjuvant therapy, and combination with immunotherapy.

#### Short course radiotherapy (SCRT)

Several studies have confirmed the practicability of SCRT. The Stockholm III study revealed that delaying surgery for 4–8 weeks after SCRT gives similar oncological results compared with SCRT with immediate surgery, as well as the long-course radiotherapy (LCRT), and did not comprise the oncological outcome [42]. In STELLAR study, 3-year DFS in SCRT group (SCRT 5 × 5 Gy → chemotherapy for 4 cycles → TME → CAPOX for 2cycles) is non-inferior to conventional CRT group (50 Gy/25 f + XELOX → TME → CAPOX for 6 cycles) as 64.5 to 62.3%, same as metastasis-free survival (MFS) and LC. Of patients who underwent re-evaluation, 11.1% in the SCRT group and 4.4% in CRT achieved cCR. The total rate of pCR and sustained cCR in the SCRT group was 21.8%, significantly higher than that of 12.3% in the CRT group [43••]. The RAPIDO trial [44] combined SCRT with TNT, aiming to reduce distant metastases without compromising local control with SCRT (5 × 5 Gy) followed by chemotherapy (CAPOX for 6 cycles or FOLFOX4 for 9 cycles) before surgery. The SCRT group came with remarkably higher pCR (28% vs 14%), lower 3-year treatment failure (23.7% vs 30.4%), and similar 3-year overall survival (89.1% vs 88.8%) compared with the standard group (50.0–50.4 Gy/25–28 f + XELOX → TME → CAPOX for 8 cycles or FOLFOX4 for 12 cycles). These studies imply that SCRT combined with neoadjuvant chemotherapy cannot only improve the therapeutic efficacy but also shorten treatment time and save medical resources, expected to be an ideal alternative to LARC.

#### Sequence of chemoradiotherapy

CAO/ARO/AIO-12 study [45] from Germany and the OPRA study [46] from USA compared the effectiveness of TNT of induction chemotherapy before neoadjuvant CRT(INCT-CRT) or consolidation chemotherapy after neoadjuvant CRT(CRT-CNCT). In CAO/ARO/AIO-12, a multicenter, randomized, phase II trial, patients with stage II-III rectal cancer were assigned to INCT-CRT or CRT-CNCT. The CRT regime was IMRT 50.4Gy/28f plus two-drug regimen (fluorouracil and oxaliplatin), the same as in the previous CAO/ARO/AIO-04 study [47] from the identical center. INCT-CRT and CRT-CNCT adopted the same scheme (oxaliplatin 100 mg/m^2^ + leucovorin 400 mg/m^2^ + fluorouracil 2400 mg/m^2^ q14d × 3 cycles). A pCR in the intention-to-treat population reached 25% in the CRT-CNCT group, fulfilling the predefined statistical hypothesis of an increased pCR in TNT compared with standard 15% after preoperative CRT. A pCR in INCT-CRT was 17% thus failed to confirm the hypothesis. CNCT group showed advantage of lower treatment-related grade 3 or 4 toxicity (27% vs 37%) and higher compliance. Surgical morbidity did not increase as the interval between CRT and surgery was prolonged in CNCT (median 90 vs 45 days in INCT group). The secondary analysis of CAO/ARO/AIO-12 showed that the two groups had similar 3-year DFS (73% in both groups) and 3-year cumulative incidence of LRR (6% vs 5%) and DM (18% vs 16%) [48••].

In the OPRA study, a prospective, randomized phase II trial, 324 rectal cancer patients with the same stage were treated by CRT-CNCT or INCT-CRT followed by either TME or WW based on tumor response [49•]. Seventy-six percent in the CRT-CNCT and 71% in the INCT-CRT group met cCR or near-complete response and were offered W&W. Two groups shared the same 3yDFS of 76% compared with 75% observed historically, however varied 3yTME-free survival of 53% and 41%, respectively. Further analysis identified minor differences in treatment compliance and adverse events between groups [49•]. It is a pity that neither of CAO/ARO/AIO-12 and OPRA studies converted higher pCR to survival benefit, but at least they propose that higher pCR of CRT-CNCT did not sacrifice oncological outcome, organ function, and QoL, providing a preferred option when given priority to organ and function preservation strategy.

#### Intensity of neoadjuvant therapy

PRODIGE 23 and PROSPECT study implies intensified chemotherapy is another option to improve pCR and survival outcome after surgery. UNICANCER-PRODIGE 23 [32•] managed to improve survival outcomes by intensifying neoadjuvant chemotherapy. In this phase 3, open-label, multicenter, randomized trial in France, 461 patients were randomly assigned to the standard-of-care group (50.4 Gy + XELOX → TME → FOLFOX for 6 months) or INCT group (FOLFIRINOX × 6 cycle → nCRT → TME → FOLFOX for 3 months). Intensified chemotherapy significantly improved pCR from 12 to 28% and 3yPFS from 62 to 69% and reduced serious adverse events during the whole treatment period. The updated report showed better long-term OS (81.9% vs 76.1%), DFS (67.6% vs 62.5%), MFS (73.6% vs 65.4%), and CSS (84.9% vs 79.6%) in the induction group. In PROSPECT [50], a non-inferiority, randomized trial of neoadjuvant FOLFOX compared with CRT, has recently been published. Untreated LARC candidates for sphincter-sparing surgery were eligible to participate. In the FOLFOX group, patients received mFOLFOX6 for 6 cycles, given CRT only if the primary tumor decreased in size by < 20% or FOLFOX was discontinued because of side effects. Of patients in the per-protocol population who underwent surgery, the percentage of patients with pCR was similar in the two groups (21.9% in the FOLFOX group and 24.3% in the CRT group). 5yOS and 5yLC were similar in the two groups (89.5% vs 90.2% and 1.8% vs 1.6% in the FOLFOX and CRT groups, respectively). While treatment without radiotherapy corresponds to CRT in short-term pathological findings and long-term oncological outcome, especially the risk of pelvic recurrence, it is worth noting that 38 patients (6.5%) treated with FOLFOX alone did not meet the clinical response threshold of a 20% decrease in primary tumor size thus taking CRT as supplementary. This implied the irreplaceability of radiotherapy for a particular population. FOWARC trial [51], in which stage II–III rectal cancer treated with fluorouracil plus radiotherapy, mFOLFOX6 plus radiotherapy, and mFOLFOX6, came with similar results. While no significant difference was found in 3yDFS (72.9%, 77.2%, 73.5%), the addition of radiotherapy improves the rate of pCR (14.0%, 27.5%, and 6.6%) and downstaging (37.1%, 56.4%, and 35.5%) [52].

#### Immunotherapy

Radiation can not only directly kill tumor cells but also modulate the immune system [53]. The combination of immunotherapy and radiotherapy in various cancers has attracted researchers’ attention [54]. Several early-phase clinical trials show superior cancer response when introducing immune checkpoint inhibitors (ICI) to conventional neoadjuvant chemoradiotherapy. Treatment regimen and cancer outcome of these trials are summarized in Table 1.

**Table 1:** Recent studies evaluating short course radiotherapy in rectal cancer

Trails	Published time	Patients	Treatment arms	pCR
VOLTAGE-A [55]	2022	cT3-4	nCRT (cape) → nivolumab × 5 → TME	30% for MSS, 60% for MSI-H
AVANA [56]	2019	cT4/N + or high-risk cT3 (CRM ≤ 1 mm, ≤ 6 cm from AV, or T3c/d)	nCRT (cape) + avelumab × 6 → TME	23%
Shamseddine et al. [57]	2020	cT2N + /cT3-4a	SCRT → mFOLFOX6 + avelumab × 6 → TME	25%
Lin et al. [46]	2021	cT3-4/N +	SCRT → CAPOX + camrelizumab × 2 → TME	48.1%, 46.2% for MSS, 100% for MSI-H

*pCR* pathologic complete response, *cape* capecitabine, *nCRT* neoadjuvant chemoradiotherapy, *TME* total mesorectal excision, *MSS* microsatellite stable, *MSI-H* high microsatellite instability, *CRM* circumferential resection margin, *AV* anal verge, *SCRT* short-course radiotherapy

In the phase I/II VOLTAGE-A trial, CRT followed by 5 cycles of nivolumab gained pCR rates of 33% in patients with microsatellite stable (MSS), exciting 60% in patients with high microsatellite instability (MSI-H) [55]. CRT followed by 6 cycles of avelumab in the phase II AVANA trial achieved pCR rate of 23% [56]. Shamseddine et al. and Lin et al. analyzed SCRT followed by consolidation chemotherapy and ICI, 25% of pCR rate and 25% of near pCR rate using 6 cycles of mFOLFOX6 plus avelumab [57], and 48.1% of pCR rate using 2 cycles of CAPOX plus camrelizumab [46]. While these studies enrolled patients without distinguishing MMR status, it is notable that patients with MSS status benefit more from immunotherapy. Exhilarating news came from 12 consecutive patients with MSS status, who achieved 100% cCR after 9 cycles of dostarlimab alone, with no recurrence, surgery, and CRT for 6–25 months [58••]. While the duration of cancer response needs longer follow-up, this outcome implies that rectal cancer patients with MSS status are highly sensitive to single PD-1 blockade and stand a good chance to exempt other therapies and better preserve organ structure and function.

For patients having received neoadjuvant therapy, oncologists adopt different strategies to achieve organ and function preservation on the basis of tumor response, general condition of patients, patient willingness, etc.

### “Watch and wait” if cCR after neoadjuvant therapy

In 2004, Habr-Gama from Brazil firstly observed that rectal cancer achieved cCR after neoadjuvant therapy is associated with excellent long-term results irrespective of treatment strategy [37]. W&W strategy was then proposed.

In primary research from Habr-Gama, 265 patients with T2-4/N + middle or distal rectal cancer were enrolled [37]. Seventy-one patients with cCR receiving nonoperative treatment were compared to 22 patients < cCR however pCR after surgery, 5yOS and 5yDFS adorable (100% vs 88%, 92% vs 83%). Mass from the Netherlands, OnCoRe from the UK, OPRA from the USA, and a study from MSKCC took the same strategy, revealing similar oncological outcome between W&W and pCR after radical surgery. The main recurrence site was the intestinal wall where the original tumor had grown, which happened within 2 years after treatment. Salvage operation worked and distal metastasis was rare [39–41, 59•]. In the ongoing prospective STAR-TREC study, patients receive SCRT or LCRT followed by W&W if cCR is reached or else transanal microsurgery [60]. The preliminary report published in ASCO2022 explained that organ preservation is safe and effective, with 60% patients reaching OP and not accumulating 24 m NRDFS. Further outcome is expected.

The W&W strategy has splendid advantages in reducing surgical trauma and preserving organ function, but also has the risk of local or distant recurrence. The IWWD-based study provided guidance on surveillance regime [61••]. By analyzing the conditional LR-free rate and DM-free rate, it was found that if cCR persists for 1 year, 3 years, and 5 years, the rates of local recurrence free in the next 2 years were 88.1%, 97.3%, and 98.6%, respectively, and the non-far-conversion rates were 93.8%, 97.8%, and 96.6%, suggesting that patients who adopted W&W after neoadjuvant therapy can appropriately reduce the monitoring intensity if they preserved cCR in the first 3 years. Implementation details for the W&W strategy need to be resolved, such as which is the better screening method for clinical evaluation and whether the intensity of neoadjuvant therapy needs to be strengthened according to the initial stage.

### Local excision for cCR or near cCR

Neoadjuvant radiotherapy or CRT shrinks tumor lesions, reduces the difficulty of surgery, and increases the possibility of sphincter preservation. A sufficient regional blood supply before surgery can improve the sensitivity of radiotherapy. Neoadjuvant therapy creates conditions for LE in patients initially unsuitable for organ preservation. Retrospective and randomized controlled trials have shown that patients after neoadjuvant radiotherapy or CRT have milder intestinal adverse reactions, a better QoL, and higher scores of multiple functions during long-term follow-up compared with patients after TME.

Lee et al. reported the oncological outcomes of 4822 patients with T2N0M0 rectal cancer who underwent radical surgery, LE followed by adjuvant CRT, or neoadjuvant CRT followed by LE between 2004 and 2014 in the US National Database [62]. The median follow-up time was 48.5 months, and the 5-year OS rates were 77.4% in the group with surgery, 76.1% in the group with adjuvant therapy, and 79.7% in the group with neoadjuvant therapy, with no statistical difference. The study suggested that CRT with LE shows comparable efficacy to radical surgery. Several prospective studies reached the same conclusion.

The ACOSOG Z6041 study [63] is a multicenter, single-arm, open-label, non-randomized phase II clinical trial. It enrolled 79 stage cT2N0 patients who received capecitabine from March 2006 to October 2009. For neoadjuvant CRT, the 3y DFS was 88.2% in the intention-to-treat arm and 86.9% in the eligible-to-treat set. A multicenter, phase II feasibility CARTS [64] study in the Netherlands included 55 patients with cT1-3N0M0 rectal cancer between February 2011 and September 2012, who received LE after a good response to neoadjuvant CRT. During the median follow-up of 53 months, 35 of 47 cases did not supplement other treatments.

A randomized study further confirmed the role of LE after neoadjuvant therapy in preserving organ function in rectal cancer. The GRECCAR 2 prospective, open-label, phase III randomized clinical trial compared LE and TME surgery in patients achieving cCR after neoadjuvant CRT for T2-3 low rectal cancer. The tumor outcomes at the 2-year follow-up did not show superiority [65]. Retrospective analysis 3 years later showed that there was no significant difference between the two groups, suggesting that LE in patients with cCR after neoadjuvant therapy can be an alternative to TME surgery [66]. No obvious superiority of LE over TME may be related to the high proportion of supplementary TME, suggesting that more precise screening of patients is needed. The recent UK TREC study [67, 68] is the first randomized clinical trial to compare neoadjuvant radiotherapy plus LE and traditional TME surgery for early-stage rectal cancer, further clarifying the feasibility of organ-sparing strategies in patients with rectal cancer ≤ T2N0M0. The study included 55 patients between February 2012 and December 2014, who were randomized 1:1 to receive TME surgery or SCRT after 8–10 weeks of LE. Those with poor prognostic factors supplement TME surgery. The primary endpoint was cumulative randomization at 12, 18, and 24 months. Among 27 patients who underwent LE after neoadjuvant radiotherapy, 8 patients (30%) achieved pCR, and 8 patients (30%) supplemented TME surgery due to poor pathological prognosis, compared with 24 patients (86%) who had poor prognosis indications in the TME surgery group. The study also included a non-randomized, prospective cohort of patients with a strong clinical indication for one treatment group. They were older than randomized patients and more likely to have life-limiting complications. Sixty-one of them received an organ-sparing strategy, of whom 24 (39%) had poor pathological prognostic indicators, and 25 patients (41%) achieved pCR. Overall, organ function preservation was achieved in 70% of randomized patients and 92% of non-randomized patients, with a limited risk of incurable local recurrence.

In terms of safety, the initial neoadjuvant CRT regimen of ACOSOG Z6041 is radiotherapy 45 Gy/1.8 Gy/5 w and extra 9 Gy for the primary tumor simultaneously combined with oral capecitabine 825 mg/m^2^ bid d1-14, d22-35 + oxaliplatin 50 mg/m^2^ d1, 2, 4, 5. Owing to treatment-related adverse reactions, the extra dose for primary tumor was reduced to 5.4 Gy, and capecitabine was reduced to 725 mg/m^2^ bid 5 days a week for 5 weeks. Adverse reactions of grade 3 or higher were 29% for gastrointestinal reactions, 15% for pain, and 15% for hematologic toxicity, compared with 4% for grade 3 or higher gastrointestinal reactions, 8% for pain, and 4% for hematologic toxicity in the radical surgery group [63]. The CARTS study found that within the follow-up period, patients receiving take organ preservation strategy experienced major, minor, and no low anterior resection syndrome at the percentage of 50%, 28%, and 22% [64]. In terms of supplementary treatment, the GRECCAR 2 study found that the more treatment methods are used, the more severe the perioperative complications may be, and the higher the risk of supplemental TME surgery owing to poor prognosis after local resection [66].

The ongoing Spanish prospective multicenter randomized controlled study TAU-TEM [69] aims to compare tumor outcomes, mortality, and quality of life between TEM and TME in patients with T2 or T3 superficial N0M0 after neoadjuvant CRT. We expect it to further expand the organ preservation in a wider range of rectal cancer patients.

## Definitive radiotherapy

Although LE with or without radiation(chemo)therapy has reduced adverse reactions, the risk of surgical complication and postoperative mortality increases with age and comorbidity. Inoperable patients are usually less tolerant to chemotherapy and turn to palliative therapy. However, some may benefit from a more curative regime using radiotherapy. Several studies evaluate the efficacy of definitive radiotherapy in elderly patients or those medically inoperable.

In a phase 1 HERBERT study [70], 38 patients with T2-4N0-1 rectal cancer, median age 83 years, received EBRT 13 × 3 Gy followed by 3 weekly high-dose-rate endorectal brachytherapy (HDREBT) boost 5–8 Gy per fraction 6 weeks later. The response occurred in 87.9% patients, with cCR 60.6%, 2y local-PFS 42%, and OS 63%. Patients with cCR showed a significant correlation with higher PFS to 60% and a trend in higher OS to 80%. Tumor volume at baseline showed a strong association with cCR, with a median volume of 10.8 cc vs. 27.3 cc in patients with or without cCR. Limited baseline tumor thickness and circumferential involvement, as well as a good response to EBRT, are associated with cCR as well. Brachytherapy shows a dose–effect relationship with most toxicity endpoints [71], but not with tumor response.

A retrospective study performed in 231 patients with cT1-2N0 distal rectal cancer showed favorable efficacy of definitive radiotherapy or CRT with cCR 58.4%, 5yOS 86.19%, PFS 83.30%, and LRFS 92.50% [72]. Patients with cCR acquired better survival compared with those with non-cCR. CRT was an independent predictor of PFS. Thus, definitive radiotherapy or CRT may be feasible in some early distal rectal cancer.

Dose–response model and systematic review demonstrated that higher dose in the range of 50.4–70.0 Gy improved tumor regression and pCR-rates and did not aggravate early toxicity for rectal cancer patients treated with consistent CRT. However, the superiority in regression did not convert to better survival outcome [73, 74]. Holliday analyzed 8408 patients of local rectal adenocarcinoma treated with definitive radiotherapy from the National Cancer Database(NCDB) in 2004–2014 [75]. Patients receiving 50.4–54 Gy had a significantly longer median, 1y and 5yOS compared with those receiving 45–50.3 Gy or > 54 Gy. A retrospective review based on NCDB by Wegner drew the same conclusion and revealed that earlier stage and increased age or comorbidity patients were more likely to receive dose escalation over 54 Gy [76]. Perhaps this frailer population had competing comorbidities leading to endpoint thus cover up the benefit of dose escalation. Notably, data from the national database had the main limitation of lack of treatment details and outcomes which could affect the efficacy of definitive radiotherapy. Besides, local control is one of the most important outcomes to track with a definitive CRT, which is not recorded either. Further randomized controlled phase 3 trials are needed to determine the best radiation dose for definitive radiotherapy.

While definitive radiotherapy provided a good tumor response, substantial risk of toxicity existed. In the HERBERT study, 68.4% and 13.2% patients experienced acute grade 2 and 3 proctitis, respectively, and 48% and 40% in late grade 2 and ≥ 3 proctitis [70]. The prescribed dose to the brachytherapy CTV(D90) was correlated with both acute and late proctitis.

## Conclusion

Organ preservation is recommended to distal rectal cancer. It omits surgical complication, preserves organ function, and thus improves the quality of life. For very early-stage patients, LE alone can achieve satisfied therapeutic effect; for patients with risky factor after LE, radiotherapy-based adjuvant therapy improves tumor outcomes; for those with advanced rectal cancer, the neoadjuvant therapy comes with higher cCR and creates opportunity for various organ preservation strategies; definitive radiotherapy with or without chemotherapy brings hope to patients inoperable or reluctant to operation. For certain individual, the best treatment plan is inconclusive, which needs to be considered in combination with the patient’s clinical characteristics, general condition, functional status, adverse reactions and recurrence risk, personal expectation and psychological tolerance, economic status and the feasibility of regular follow-up, and other factors. A multidisciplinary comprehensive evaluation is of great significance. A subtle follow-up design will provide researchers with a closer observation for changes in patients’ condition and be able to treat timely. Deep communication about benefits and risks of each treatment option with patients and their family members also matters. Then, finally, can we decide the treatment strategies on the basis of organ preservation. More data from prospective and multicenter studies are needed to confirm the non-inferiority of organ preservation strategy for distal rectal cancer in terms of cancer control and function reservation versus standard treatment before this strategy can be recommended more widely.

## Data Availability

No datasets were generated or analysed during the current study.
